# Supplementary material for: The sponge microbiome within the greater coral reef microbial metacommunity
Source: Nat Commun. 2019 Apr 9;10:1644. doi: 10.1038/s41467-019-09537-8 (PMC6456735; doi:10.1038/s41467-019-09537-8)
Supplement: Supplementary file 1 — Supplementary Information [file 41467_2019_9537_MOESM1_ESM.pdf]

The sponge microbiome within the greater coral reef microbial metacommunity

Cleary et al.

Supplementary Table 1.

Supplementary Figure 1.

Supplementary Figure 2.

Supplementary Figure 3.

Supplementary Figure 4.

Supplementary Figure 5.

Supplementary Figure 6.

Supplementary Figure 7.

Phylum	Class	Sequences	OTUs
Proteobacteria		1191455	13592
	Gammaproteobacteria	573036	5815
	Alphaproteobacteria	422132	2086
	Deltaproteobacteria	93637	5187
	Betaproteobacteria	67233	204
	Epsilonproteobacteria	9132	58
	ARKDMS-49	8893	20
Bacteroidetes		159220	4290
Chloroflexi		131590	839
Tenericutes		131156	191
Actinobacteria		101587	571
Cyanobacteria		79405	437
Acidobacteria		54725	1236
Spirochaetae		45664	427
Planctomycetes		43093	3469
Thaumarchaeota		31926	60
Nitrospirae		27075	141
Gemmatimonadetes		23719	416
Euryarchaeota		21322	100
Verrucomicrobia		21190	886
Tectomicrobia		11771	81
Chlamydiae		11373	324
Firmicutes		11333	520
PAUC34f		9535	69
SBR1093		9497	46
Parcubacteria		7173	386
Poribacteria		6132	58
Lentisphaerae		5012	138
Marinimicrobia (SAR406 clade)		4073	69
Nitrospinae		3218	55
Deinococcus-Thermus		3194	28
Latescibacteria		2657	214
Fusobacteria		2230	36
Woesearchaeota (DHVEG-6)		2152	764
Saccharibacteria		1875	168
Deferribacteres		1597	80
Gracilibacteria		928	79
Peregrinibacteria		449	82
Fibrobacteres		401	103
Chlorobi		388	37
Elusimicrobia		340	77
Hydrogenedentes		330	66
Omnitrophica		313	185
BRC1		125	63
Ignavibacteriae		107	32
SR1 (Absconditabacteria)		81	22
TM6 (Dependentiae)		78	68
Armatimonadetes		62	8
WA-aaa01f12		60	12
KSB3 (Modulibacteria)		49	24
Aminicenantes		38	16
RBG-1 (Zixibacteria)		38	20
WS2		36	10
uncultured archaeon		31	7
Bathyarchaeota		30	18
Acetothermia		29	8
Marine Hydrothermal Vent Group(MHVG)		25	12
Aenigmarchaeota		19	16
FCPU426		17	9
WWE3		13	10
LCP-89		10	8
Lokiarchaeota		8	8
Candidatus Berkelbacteria		7	7
Microgenomates		7	6
FL0428B-PF49		6	2
Synergistetes		5	2
Cloacimonetes		4	4
Ancient Archaeal Group(AAG)		3	2
WSA2		3	2
AC1		2	1
Altiarchaeales		2	1
Diapherotrites		2	2
BJ-169		1	1
Hadesarchaea		1	1
Marine Benthic Group E		1	1
Miscellaneous Euryarchaeotic Group(MEG)		1	1
WS6		1	1

Supplementary Table 1. Number of sequences and OTUs recorded for all phyla and the major proteobacterial classes in the present study based on the Silva 128 database.

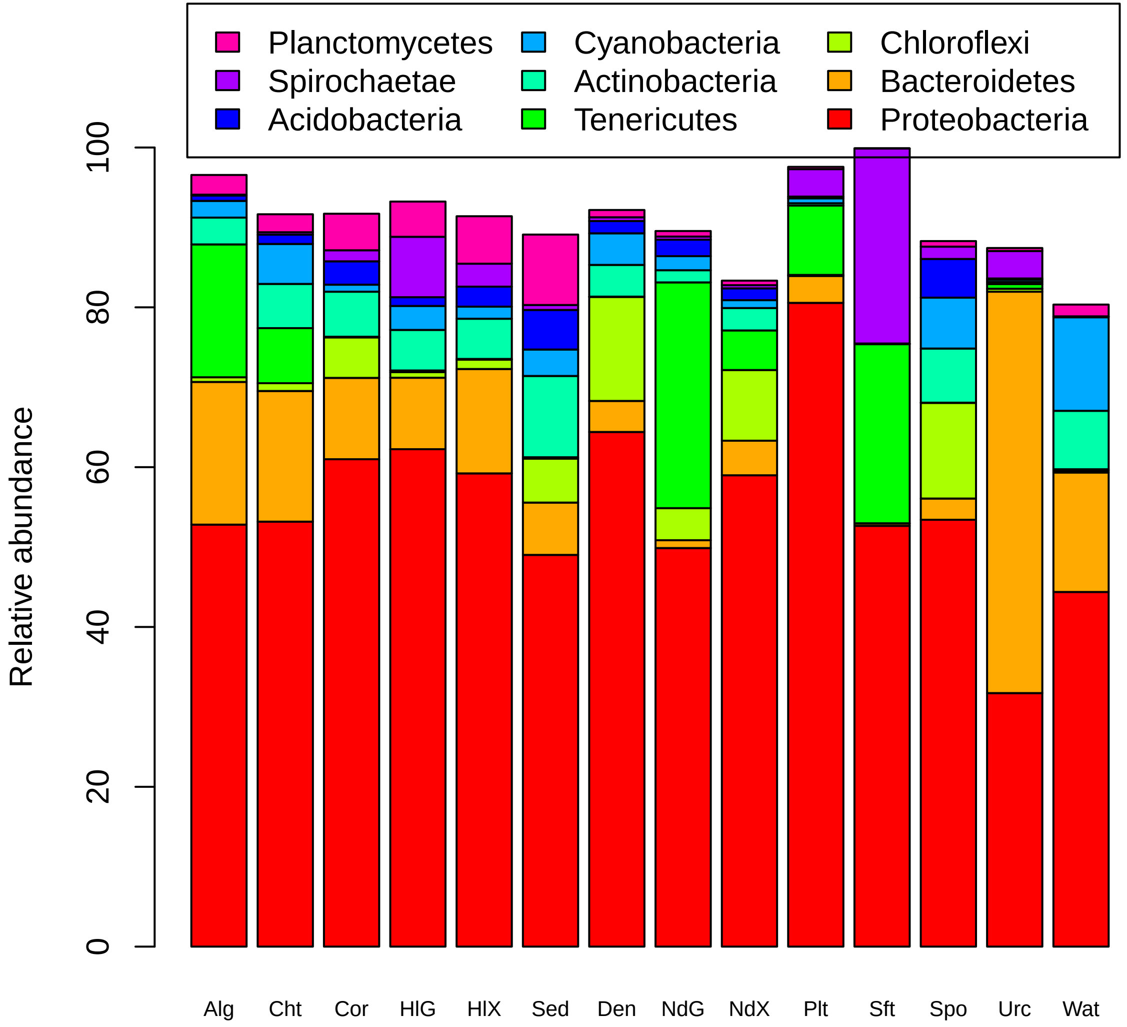

Supplementary Fig. 1: Stacked barplot of the relative abundance of the nine most abundant phyla in algae (Alg), chitons (Cht), stony corals (Cor), sea cucumber gut (HlG), sea cucumber mantle (HlX), flatworms (Plt), sediment (Sed), sponges (Spo), sponge denizens (Den), nudibranch gut (NdG), nudibranch mantle (NdX), flatworms (Plt), soft corals (Sft), sponges (Spo), sea urchins (Urc) and seawater (Wat). Source data are provided as a Source Data file.

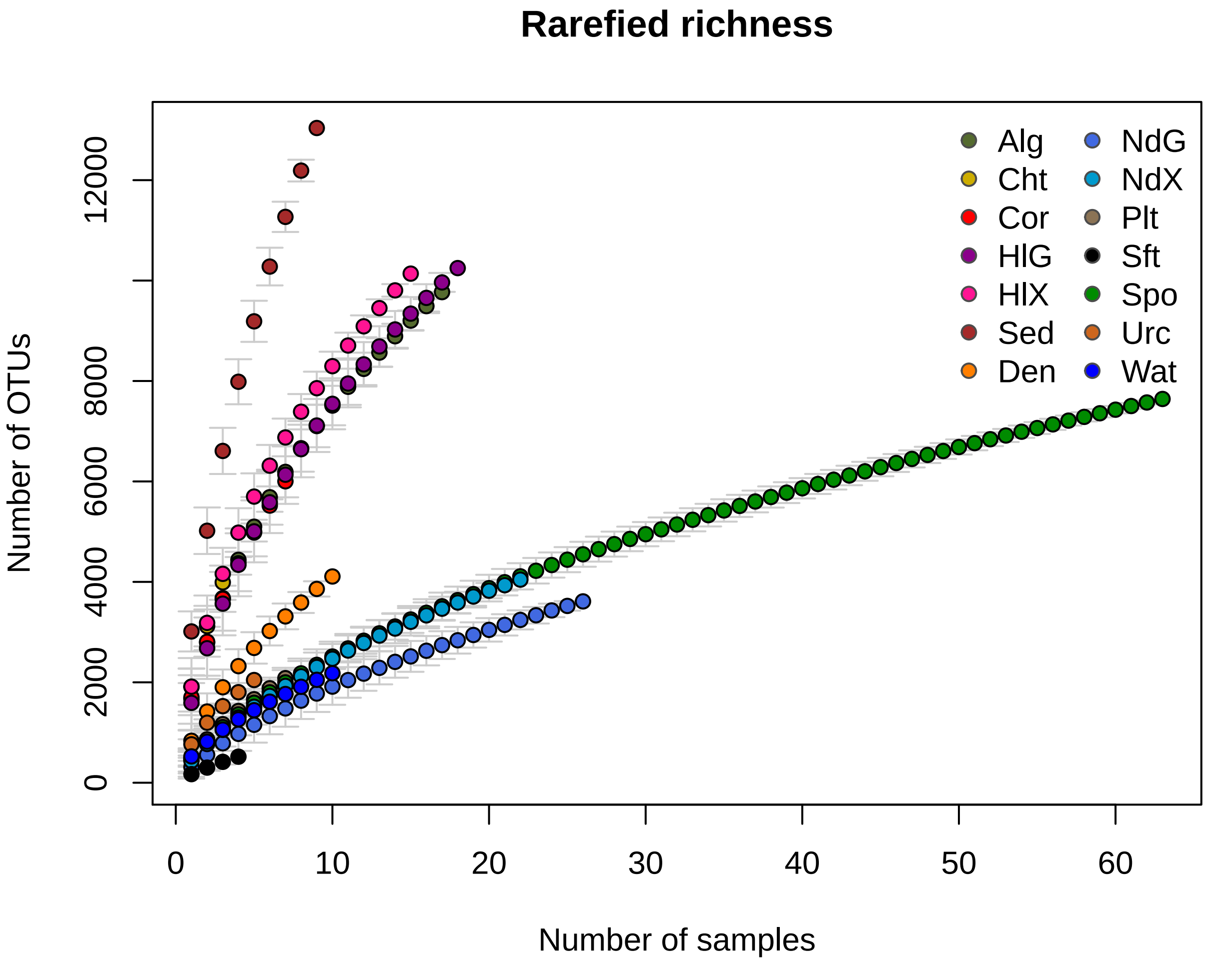

Supplementary Fig. 2. Cumulative OTU richness (error bars represent 95% confidence intervals) of algae (Alg), chitons (Cht), stony corals (Cor), sea cucumber gut (HlG), sea cucumber mantle (HlX), flatworms (Plt), sediment (Sed), sponges (Spo), sponge denizens (Den), nudibranch gut (NdG), nudibranch mantle (NdX), flatworms (Plt), soft corals (Sft), sponges (Spo), sea urchins (Urc) and seawater (Wat) estimated using the specaccum function in vegan with the ‘method’ argument set to ‘random’ and 999 permutations . Error bars represent a single standard deviation. Each sample represents 10000 sequences. Source data are provided as a Source Data file.

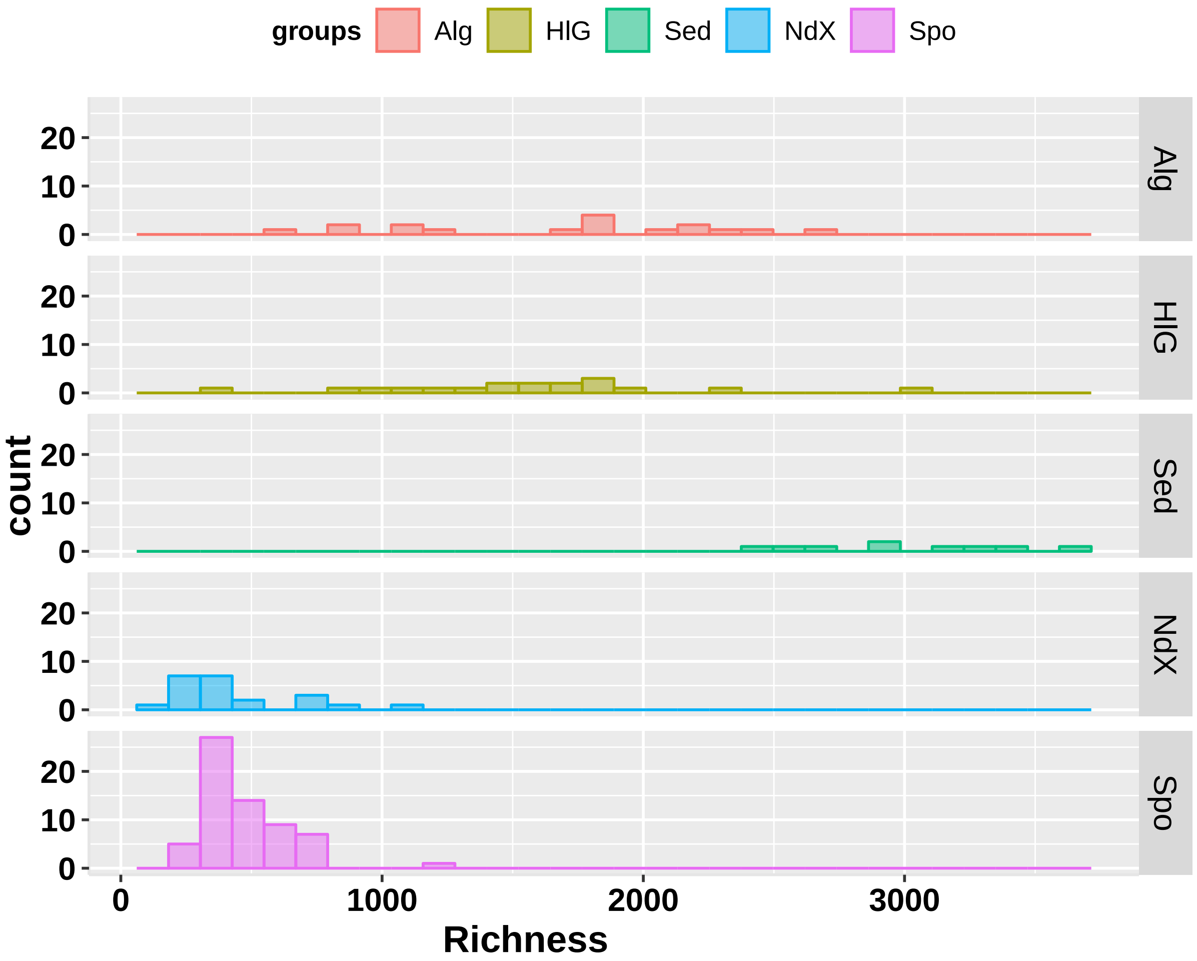

Supplementary Fig. 3. Histograms of richness counts for samples of algae (Alg), sea cucumber gut (HlG), sediment (Sed), sponges (Spo) and the nudibranch mantle (NdX) made with the ggplot2.histogram function in the easyGgplot2 library in R. Source data are provided as a Source Data file.

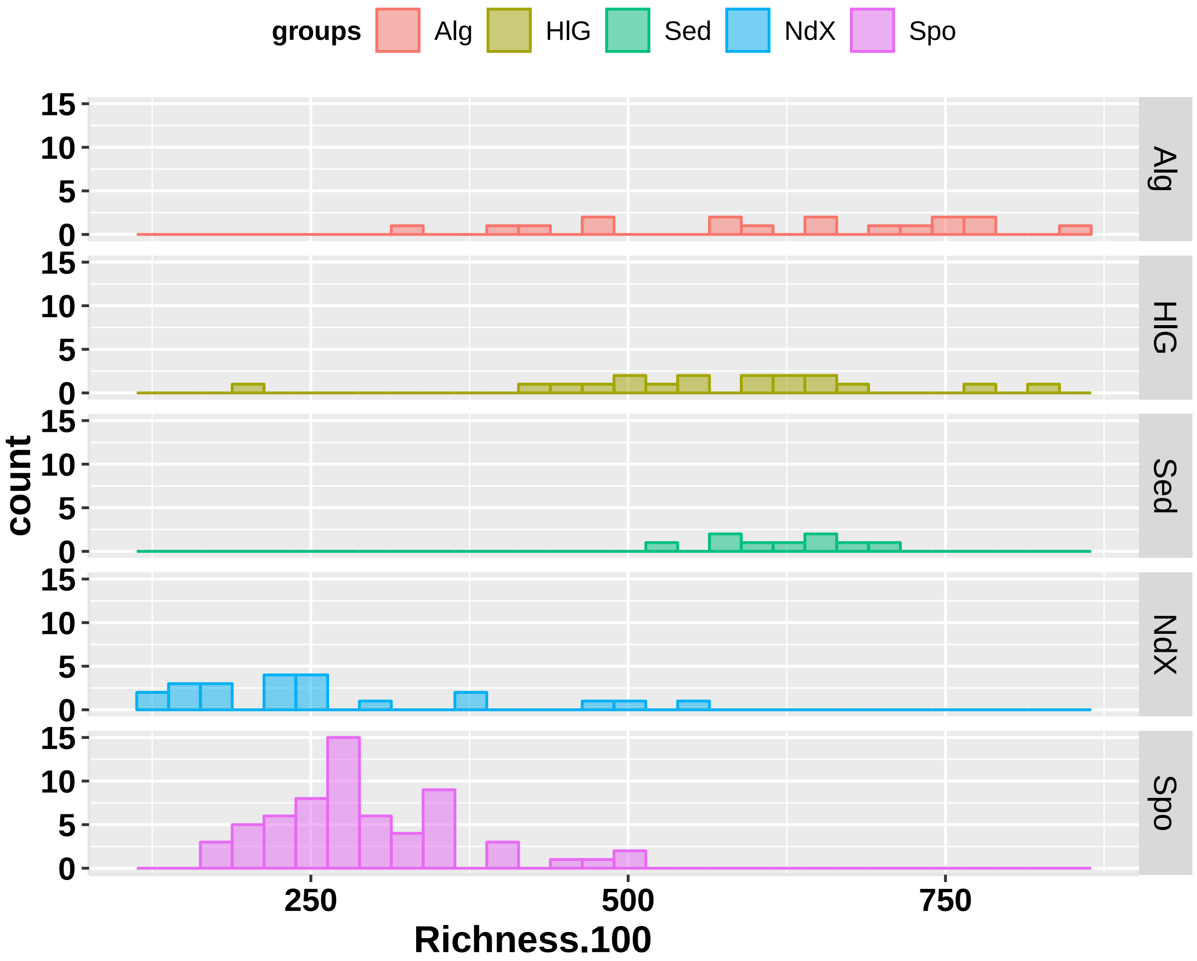

Supplementary Fig. 4. Histograms of richness counts, only including all OTUs > 100 sequences (OTUs100), for samples of algae (Alg), sea cucumber gut (HIG), sediment (Sed), sponges (Spo) and the nudibranch mantle (NdX) made with the ggplot2.histogram function in the easyGgplot2 library in R. Source data are provided as a Source Data file.

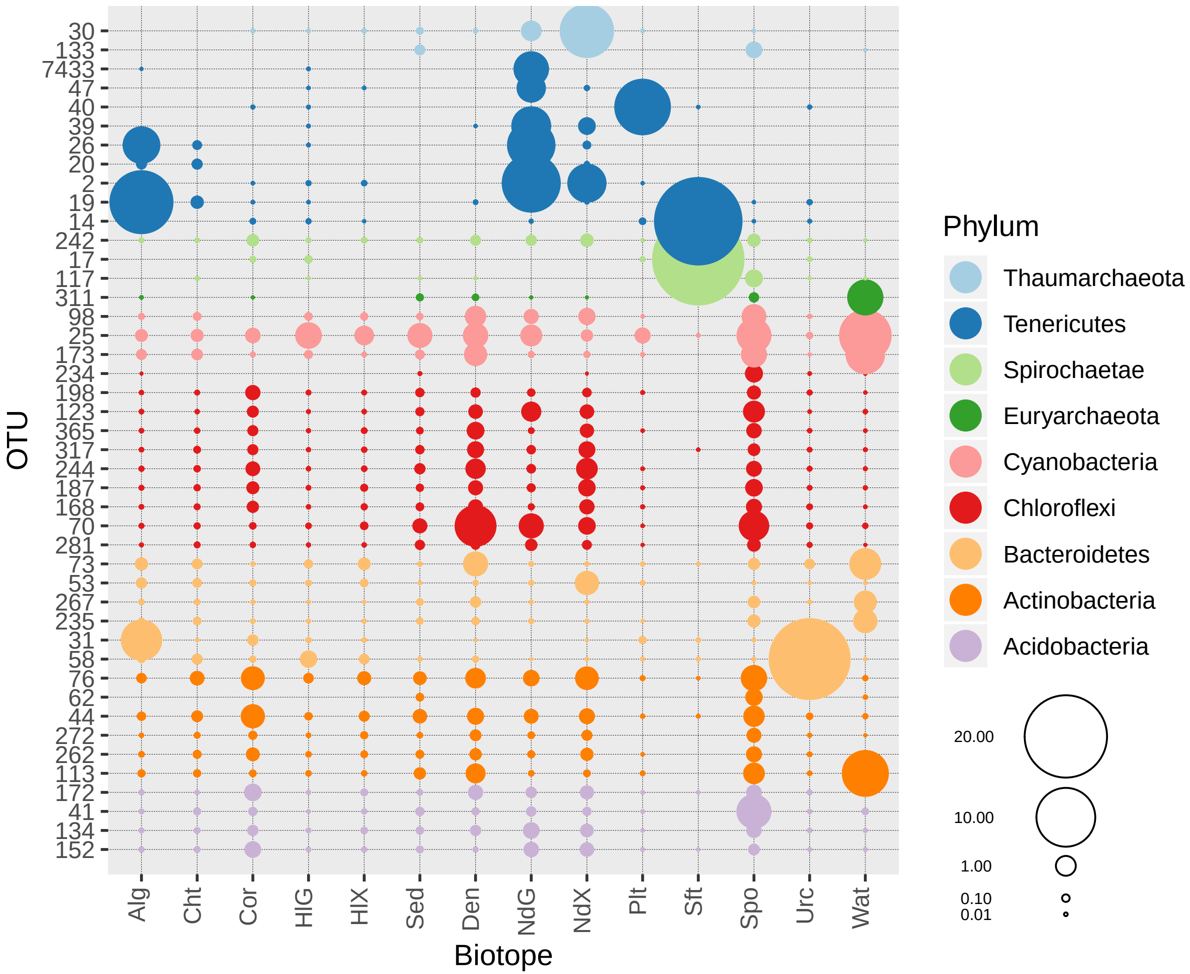

Supplementary Fig. 5. Relative abundance of the most abundant OTUs, colour-coded according to prokaryote phyla, in algae (Alg), chitons (Cht), stony corals (Cor), sea cucumber gut (HlG), sea cucumber mantle (HlX), flatworms (Plt), sediment (Sed), sponges (Spo), sponge denizens (Den), nudibranch gut (NdG), nudibranch mantle (NdX), flatworms (Plt), soft corals (Sft), sponges (Spo), sea urchins (Urc) and seawater (Wat). The phyla included are: Acidobacteria, Actinobacteria, Bacteroidetes, Chloroflexi, Cyanobacteria, Euryarchaeota, Spirochaetae, Tenericutes and Thaumarchaeota. The size of the symbol is proportional to the relative abundance of sequences represented by a given OTU. Source data are provided as a Source Data file.

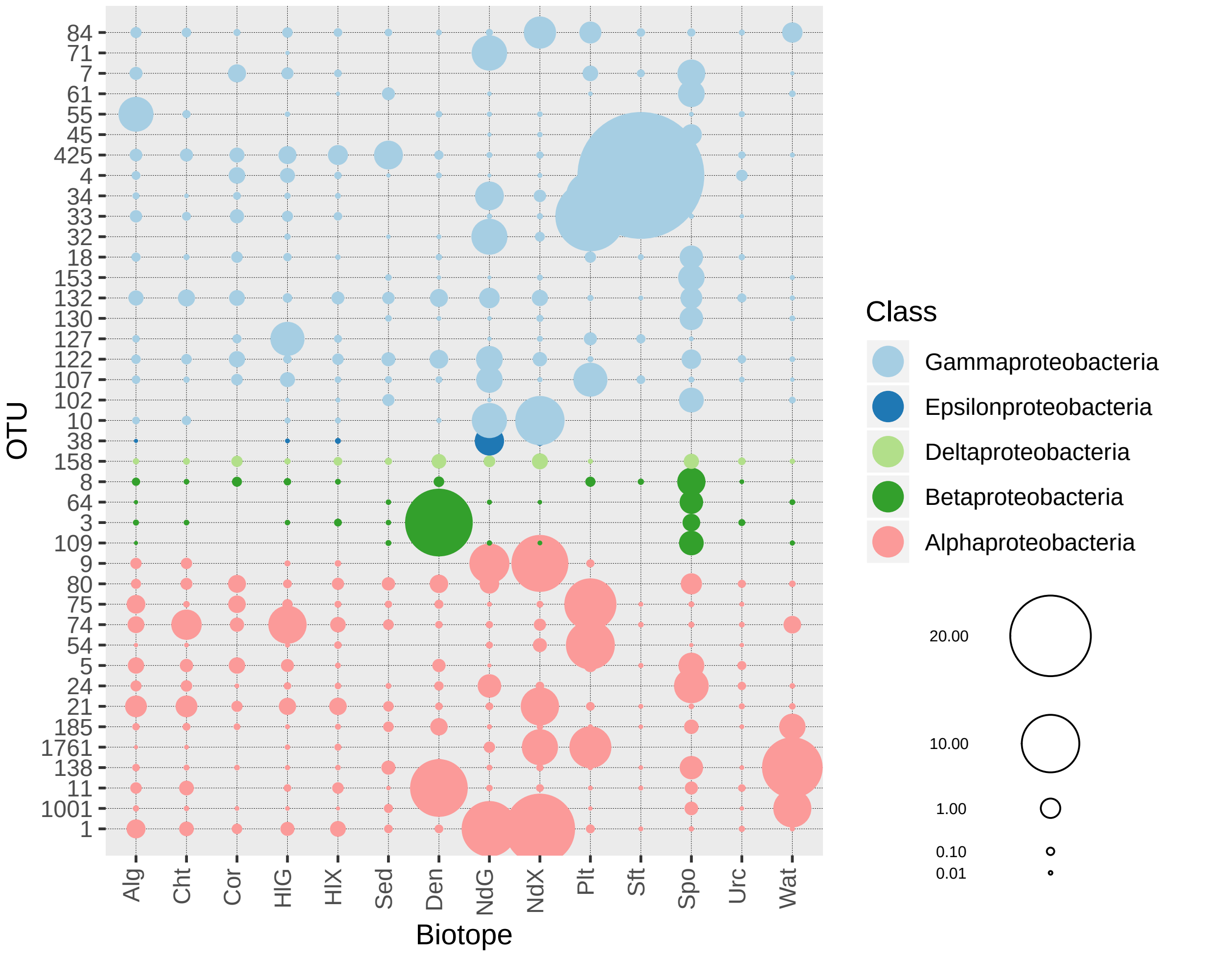

Supplementary Fig. 6. Relative abundance of the most abundant OTUs, colour-coded according to proteobacterial class, in algae (Alg), chitons (Cht), stony corals (Cor), sea cucumber gut (HlG), sea cucumber mantle (HlX), flatworms (Plt), sediment (Sed), sponges (Spo), sponge denizens (Den), nudibranch gut (NdG), nudibranch mantle (NdX), flatworms (Plt), soft corals (Sft), sponges (Spo), sea urchins (Urc) and seawater (Wat). The classes included are: Alphaproteobacteria, Betaproteobacteria, Deltaproteobacteria, Epsilonproteobacteria and Gammaproteobacteria. The size of the symbol is proportional to the relative abundance of sequences represented by a given OTU. The y-axis shows the OTU id number. Source data are provided as a Source Data file.

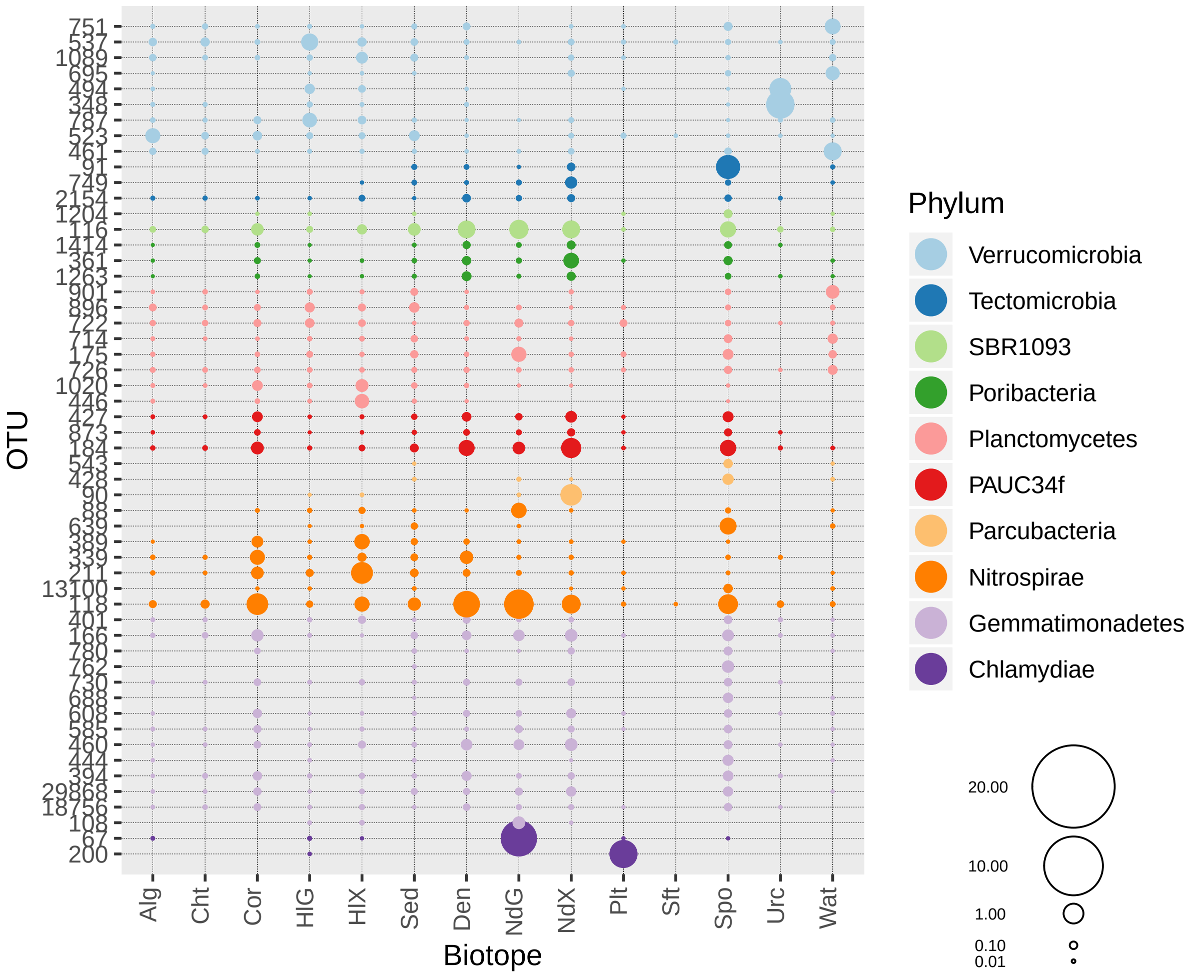

Supplementary Fig. 7. Relative abundance of OTUs, colour-coded according to prokaryote phyla, in algae (Alg), chitons (Cht), stony corals (Cor), sea cucumber gut (HlG), sea cucumber mantle (HlX), flatworms (Plt), sediment (Sed), sponges (Spo), sponge denizens (Den), nudibranch gut (NdG), nudibranch mantle (NdX), flatworms (Plt), soft corals (Sft), sponges (Spo), sea urchins (Urc) and seawater (Wat). The phyla included are: Chlamydiae, Gemmatimonadetes, Nitrospirae, Parcubacteria, PAUC34f, Planctomycetes, Poribacteria, SBR1093, Tectomicrobia and Verrucomicrobia. The size of the symbol is proportional to the relative abundance of sequences represented by a given OTU. The y-axis shows the OTU id number. Source data are provided as a Source Data file.
