# Supplementary material for: The sponge microbiome within the greater coral reef microbial metacommunity
Source: Nat Commun. 2019 Apr 9;10:1644. doi: 10.1038/s41467-019-09537-8 (PMC6456735; doi:10.1038/s41467-019-09537-8)
Supplement: Supplementary file 3 — Reporting Summary [file 41467_2019_9537_MOESM3_ESM.pdf]

nature research

Corresponding author(s): Daniel FR Cleary

Last updated by author(s): Mar 15, 2019

Reporting Summary

Nature Research wishes to improve the reproducibility of the work that we publish. This form provides structure for consistency and transparency in reporting. For further information on Nature Research policies, see Authors & Referees and the Editorial Policy Checklist.

Statistics

For all statistical analyses, confirm that the following items are present in the figure legend, table legend, main text, or Methods section.

n/a	Confirmed	
☐	☒	The exact sample size (n) for each experimental group/condition, given as a discrete number and unit of measurement
☐	☒	A statement on whether measurements were taken from distinct samples or whether the same sample was measured repeatedly
☐	☒	The statistical test(s) used AND whether they are one- or two-sided<br>*Only common tests should be described solely by name; describe more complex techniques in the Methods section.*
☒	☐	A description of all covariates tested
☐	☒	A description of any assumptions or corrections, such as tests of normality and adjustment for multiple comparisons
☐	☒	A full description of the statistical parameters including central tendency (e.g. means) or other basic estimates (e.g. regression coefficient) AND variation (e.g. standard deviation) or associated estimates of uncertainty (e.g. confidence intervals)
☐	☒	For null hypothesis testing, the test statistic (e.g. F, t, r) with confidence intervals, effect sizes, degrees of freedom and P value noted<br>*Give P values as exact values whenever suitable.*
☒	☐	For Bayesian analysis, information on the choice of priors and Markov chain Monte Carlo settings
☒	☐	For hierarchical and complex designs, identification of the appropriate level for tests and full reporting of outcomes
☒	☐	Estimates of effect sizes (e.g. Cohen's d, Pearson's r), indicating how they were calculated

Our web collection on statistics for biologists contains articles on many of the points above.

Software and code

Policy information about availability of computer code

Data collection	NA
Data analysis	R, QIIME, AWK, PYTHON

For manuscripts utilizing custom algorithms or software that are central to the research but not yet described in published literature, software must be made available to editors/reviewers. We strongly encourage code deposition in a community repository (e.g. GitHub). See the Nature Research guidelines for submitting code & software for further information.

Data

Policy information about availability of data

All manuscripts must include a data availability statement. This statement should provide the following information, where applicable:

- Accession codes, unique identifiers, or web links for publicly available datasets
- A list of figures that have associated raw data
- A description of any restrictions on data availability

The DNA sequences generated in this study can be downloaded from NCBI BioProject Ids: PRJNA382576, PRJNA397173, PRJNA397177, PRJNA397178.

Field-specific reporting

Please select the one below that is the best fit for your research. If you are not sure, read the appropriate sections before making your selection.

☐ Life sciences ☐ Behavioural & social sciences ☒ Ecological, evolutionary & environmental sciences

For a reference copy of the document with all sections, see nature.com/documents/nr-reporting-summary-flat.pdf

Ecological, evolutionary & environmental sciences study design

All studies must disclose on these points even when the disclosure is negative.

Study description	Specimens were sampled in various reef sites in Taiwan and Thailand. The primary goal was to assess prokaryote composition in multiple host-associated and environmental (sediment and seawater) biotopes.
Research sample	Environmental samples from various marine hosts, sediment and filters through which seawater had been pumped.
Sampling strategy	We strove to sample at least three specimens for each species across multiple sampling sites.
Data collection	Samples were collected by SCUBA diving with a focus on particular host taxa including sponges, sea cucumbers and other taxa.
Timing and spatial scale	Sampling took place from 2014-07-22 to 2014-08-21 in Thailand and 2016-07-20 to 2016-08-07 in Taiwan. All samples from Thailand were collected from sites around Koh Tao island, Phuket island and close to Pattaya. All samples from Taiwan were collected from the Penghu islands.
Data exclusions	All samples with less than 10000 sequence reads, after quality control, were excluded from analysis.
Reproducibility	The study was a field survey
Randomization	Samples were collected haphazardly
Blinding	The study was a field survey aiming to study differences in prokaryote composition among host taxa. It was not an experiment where blinding would be appropriate.

Did the study involve field work? ☒ Yes ☐ No

Field work, collection and transport

Field conditions	Annual variation of mean sea surface temperature ranged from 21 to 28 °C in Taiwan and 27 to 31 °C in Thailand. All sample sites were coral reef habitat with varying degrees of coral cover.
Location	Sampling took place from 2014-07-22 to 2014-08-21 in Thailand and 2016-07-20 to 2016-08-07 in Taiwan.
Access and import/export	All samples were collected in compliance with national laws and scientists who took part in and enabled sampling in Taiwan and Thailand are also coauthors of the manuscript.
Disturbance	Disturbance was minimal and included sampling small portions of host taxa, sediment and one liter samples of sea water.

Reporting for specific materials, systems and methods

We require information from authors about some types of materials, experimental systems and methods used in many studies. Here, indicate whether each material, system or method listed is relevant to your study. If you are not sure if a list item applies to your research, read the appropriate section before selecting a response.

Materials & experimental systems

n/a	Involved in the study	
☒	☐	Antibodies
☒	☐	Eukaryotic cell lines
☒	☐	Palaeontology
☐	☒	Animals and other organisms
☒	☐	Human research participants
☒	☐	Clinical data

Methods

n/a	Involved in the study	
☒	☐	ChIP-seq
☒	☐	Flow cytometry
☒	☐	MRI-based neuroimaging

Animals and other organisms

Policy information about studies involving animals; ARRIVE guidelines recommended for reporting animal research

Laboratory animals	No laboratory animals were used for this study.
Wild animals	Numerous animals were sampled for this study, which included 216 samples. For most animals, e.g., sponges, only a portion of the animal was sampled. For other animals, e.g., nudibranchs and flatworms, the whole organism was sampled. Care was taken to perturb the natural environment as little as possible.

Field-collected samples	All samples were cooled as soon as possible in the field by storing them in a cooler filled with ice. Once in the laboratory, samples were stored at -20 °C and DNA was extracted as soon as possible. Sample collection and preparation follows all of our previous studies whereby samples were located in the field and photographed with a collection number that was specific for each expedition/field work period. Samples were then stored underwater in a plastic bag with the collection number. Once back at the field station, samples were prepared, photographed again and stored in alcohol or other preservation agents depending on the research question.
Ethics oversight	No ethical guidance or approval was required or necessary. All fieldwork was carried out with local host scientists.

Note that full information on the approval of the study protocol must also be provided in the manuscript.
