# Supplementary material for: The sponge microbiome within the greater coral reef microbial metacommunity
Source: Nat Commun. 2019 Apr 9;10:1644. doi: 10.1038/s41467-019-09537-8 (PMC6456735; doi:10.1038/s41467-019-09537-8)
Supplement: Supplementary file 4 — Description of Additional Supplementary Files [file 41467_2019_9537_MOESM4_ESM.pdf]

Description of Additional Supplementary Files

File Name: Supplementary Data 1
Description: List of samples used in the present study including the sample code (Sample), biotope code (Bio), Biotope, Species, sampling date (Date), sampling site (Site), Country, Depth, Latitude (Lat), Longitude (Lon) and location (Country). The percentages of the most abundant phyla, classes and orders are given as is the dominance (Dom; relative abundance of the most abundant OTU), Dom3 (relative abundance of the three most abundant OTUs), rarefied richness (Richness), Pielou's J (J), Shannon's H' diversity index (H), coordinates for the PCO ordination (PC1, PC2, PC3 and PC4), number of OTUs100 shared with sediment only (SdOTUs), number of OTUs100 shared with seawater only (WtOTUs), number of OTUs100 shared with sediment and/or seawater (EnOTUs), percentage of OTUs100 shared with sediment only (SdOTUs%), percentage of OTUs100 shared with seawater only (WtOTUs%), percentage of OTUs100 shared with sediment and/or seawater (EnOTUs%), number of sequences shared with sediment only (SdSeqs), number of sequences shared with seawater only (WtSeqs), number of sequences shared with sediment and/or seawater only (EnSeqs), percentage of sequences shared with sediment only (SdSeqs%), percentage of sequences shared with seawater only (WtSeqs%) and percentage of sequences shared with sediment and/or seawater (EnSeqs%).

File Name: Supplementary Data 2
Description: Results of emmeans analysis showing pairwise comparisons of differences in the relative abundances of selected phyla, classes and the number of OTUs100 shared with sediment only (SdOTUs), number of OTUs100 shared with seawater only (WtOTUs), number of OTUs100 shared with sediment and/or seawater (EnOTUs), percentage of OTUs100 shared with sediment only (SdOTUs%), percentage of OTUs100 shared with seawater only (WtOTUs%), percentage of OTUs100 shared with sediment and/or seawater (EnOTUs%), number of sequences shared with sediment only (SdSeqs), number of sequences shared with seawater only (WtSeqs), number of sequences shared with sediment and/or seawater only (EnSeqs), percentage of sequences shared with sediment only (SdSeqs%), percentage of sequences shared with seawater only (WtSeqs%) and percentage of sequences shared with sediment and/or seawater (EnSeqs%) between biotopes based on the 'fdr' test. Significance: * 0.01 < Pr < 0.05 ** 0.001< Pr < 0.01; *** Pr < 0.001. Variable: the dependent variable, Contrast: Contrasts between pairs of biotopes, SE: standard error.

File Name: Supplementary Data 3
Description: Results of Simper analysis showing the contribution of OTUs to differences in similarity between pairs of biotopes. Contrast: contrasts between pairs of biotopes. Average: average contribution to overall dissimilarity. Sd: Standard deviation of contribution. Ratio: Average to sd ratio. AvA, AvB: average abundances per biotope. CumSum: ordered cumulative contribution. P: permutation p-value. OTUs that contributed significantly to differences are indicated by significance (Sig.): * 0.01 < P < 0.05 ** 0.001< P < 0.01; *** P < 0.001.

File Name: Supplementary Data 4
Description: List of most abundant OTUs and closely related organisms identified using BLAST search. OTU: OTU id number; Sum: number of sequence reads; Acc: Genbank accession numbers of closely related organisms identified using BLAST; Seq: sequence similarities of these organisms with our representative OTU sequences; Source: isolation source of organisms identified using BLAST.

File Name: Supplementary Data 5
Description: List of all OTUs with > 100 sequences. Shared biotopes (number of biotopes in which the OTUs in question were recorded) and Abund (number of sequence reads) for algae (Alg), chitons (Cht), stony corals (Cor), sea cucumber gut (HlG), sea cucumber mantle (HlX), flatworms (Plt),

sediment (Sed), sponges (Spo), sponge denizens (Den), nudibranch gut (NdG), nudibranch mantle (NdX), flatworms (Plt), soft corals (Sft), sponges (Spo), sea urchins (Urc) and seawater (Wat).
